# Precise Temperature Compensation of Phase in a Rhythmic Motor Pattern

**DOI:** 10.1371/journal.pbio.1000469

**Published:** 2010-08-31

**Authors:** Lamont S. Tang, Marie L. Goeritz, Jonathan S. Caplan, Adam L. Taylor, Mehmet Fisek, Eve Marder

**Affiliations:** Volen Center and Biology Department, Brandeis University, Waltham, Massachusetts, United States of America; University of Cambridge, United Kingdom

## Abstract

Most animal species are cold-blooded, and their neuronal circuits must maintain function despite environmental temperature fluctuations. The central pattern generating circuits that produce rhythmic motor patterns depend on the orderly activation of circuit neurons. We describe the effects of temperature on the pyloric rhythm of the stomatogastric ganglion of the crab, *Cancer borealis*. The pyloric rhythm is a triphasic motor pattern in which the Pyloric Dilator (PD), Lateral Pyloric (LP), and Pyloric (PY) neurons fire in a repeating sequence. While the frequency of the pyloric rhythm increased about 4-fold (Q_10_∼2.3) as the temperature was shifted from 7°C to 23°C, the phase relationships of the PD, LP, and PY neurons showed almost perfect temperature compensation. The Q_10_'s of the input conductance, synaptic currents, transient outward current (I_A_), and the hyperpolarization-activated inward current (I_h_), all of which help determine the phase of LP neuron activity, ranged from 1.8 to 4. We studied the effects of temperature in >1,000 computational models (with different sets of maximal conductances) of a bursting neuron and the LP neuron. Many bursting models failed to monotonically increase in frequency as temperature increased. Temperature compensation of LP neuron phase was facilitated when model neurons' currents had Q_10_'s close to 2. Together, these data indicate that although diverse sets of maximal conductances may be found in identified neurons across animals, there may be strong evolutionary pressure to restrict the Q_10_'s of the processes that contribute to temperature compensation of neuronal circuits.

## Abstract 2

Computational modeling and experimentation in a model system for network dynamics reveal how network phase relationships are temperature-compensated in terms of their underlying synaptic and intrinsic membrane currents.

## Author Summary

The neural circuits that produce behaviors such as walking, chewing, and swimming must be both robust and flexible to changing internal and environmental demands. How then do cold-blooded animals cope with temperature fluctuations when the underlying processes that give rise to circuit performance are themselves temperature-dependent? We exploit the crab stomatogastric ganglion to understand the extent to which circuit features are robust to temperature perturbations. We subjected these circuits to temperature ranges they normally encounter in the wild. Interestingly, while the frequency of activity in the network increased 4-fold over these temperature ranges, the relative timing between neurons in the network—termed phase relationships—remained constant. To understand how temperature compensation of phase might occur, we characterized the temperature dependence (Q_10_'s) of synapses and membrane currents. We used computational models to show that the experimentally measured Q_10_'s can promote phase maintenance. We also showed that many model bursting neurons fail to burst over the entire temperature range and that phase maintenance is promoted by closely restricting the model neurons' Q_10_'s. These results imply that although ion channel numbers can vary between individuals, there may be strong evolutionary pressure that restricts the temperature dependence of the processes that contribute to temperature compensation of neuronal circuits.

## Introduction

The nervous systems of cold-blooded animals must function across significant ranges of temperature despite the fact that the signal transduction pathways and synaptic and intrinsic membrane currents are all temperature-dependent. This is particularly intriguing because one would not expect all cellular processes to have the same temperature dependence, and therefore it is hard to imagine that functional circuit integrity would necessarily be easily maintained when temperature is altered. Nonetheless, temperature compensation, that is maintenance of constant function as temperature is altered, is an important property of circadian rhythms [1],[2] and has been reported in other systems as well [3].

All biological processes have characteristic Q_10_'s that describe the changes in their rates as a function of temperature. Although the Q_10_ for many biological processes is ∼2, Q_10_'s for ion channels can vary from 1.5 to almost 100. For example, the Q_10_'s for activation and inactivation of some K^+^ channels are 1.8–4.6 [4], many temperature sensing TRP channels have Q_10_'s higher than 10 [5], and some Ca^2+^ channels have inactivation rates with Q_10_'s as high as 19 [6]. How different can the various Q_10_'s that govern the intrinsic and synaptic conductances within a circuit be and still allow appropriate function to be maintained despite environmental temperature change? Which attributes of circuit performance are temperature dependent and which, if any, are temperature compensated?

*Cancer borealis*, the crab used for this study, lives in the northern Atlantic Ocean, and can be found from Newfoundland to Florida [7],[8],[9]. While most *C. borealis* inhabit deeper waters (at depths of 800 m), they also are frequently found in both intertidal and subtidal ecosystems foraging for food [7],[8],[9],[10],[11]. During the summer at these shallow depths (ranging from 0–10 m), *C. borealis* can experience temperature fluctuations ranging from 8°C to 24°C within a single day [10],[11]. During the winter, *C. borealis* is found at ocean temperatures ranging from 3°C to 16°C [8]. Presumably, the nervous system of these animals can cope with such considerable temperature fluctuations.

The stomatogastric ganglion (STG) of marine crustaceans generates two motor patterns that are responsible for the chewing and filtering of food [12]. The pyloric rhythm is a triphasic motor pattern driven by a three-neuron pacemaker ensemble (the single Anterior Burster (AB) and two Pyloric Dilator (PD) neurons). The Lateral Pyloric (LP) and Pyloric (PY) neurons fire on rebound from inhibition by the pacemaker neurons. Previous work has indicated that a number of processes can influence the phase of the LP and PY neurons' rebound firing. These include the strength and time course of the inhibitory synapses that the LP and PY neurons receive from the AB and PD neurons [13],[14],[15] and the conductances of the transient outward K^+^ current (I_A_) and the hyperpolarization-activated inward current (I_h_) [16],[17],[18].

The phase relationships of the network neurons are maintained relatively constant as a function of frequency [15],[19],[20],[21],[22],[23] and during the animal's growth [19],[24]. This phase constancy over a range of frequencies has been extensively studied in preparations held at constant temperature. We now show that although temperature drastically alters the frequency of the pyloric rhythm, its phase relationships are remarkably temperature invariant. This motivated us to examine the effects of temperature on the synaptic and intrinsic membrane currents that have been previously implicated in the control of phase in the pyloric rhythm. By so doing, we attempt to account for the temperature compensation of pyloric rhythm phase in terms of the effects of temperature on some of its membrane conductances.

Although the pyloric rhythm is a “simple” neuronal circuit, its dynamics involve the activation and inactivation of many intrinsic and synaptic currents. To determine whether the biological results “automatically” arise from the effects of temperature on membrane currents with similar Q_10_'s, we varied temperature in two different computational models, one of a bursting pacemaker neuron [25] and one of the LP neuron [26].

## Results

### Effects of Temperature on the Pyloric Rhythm

**Figure 1:**
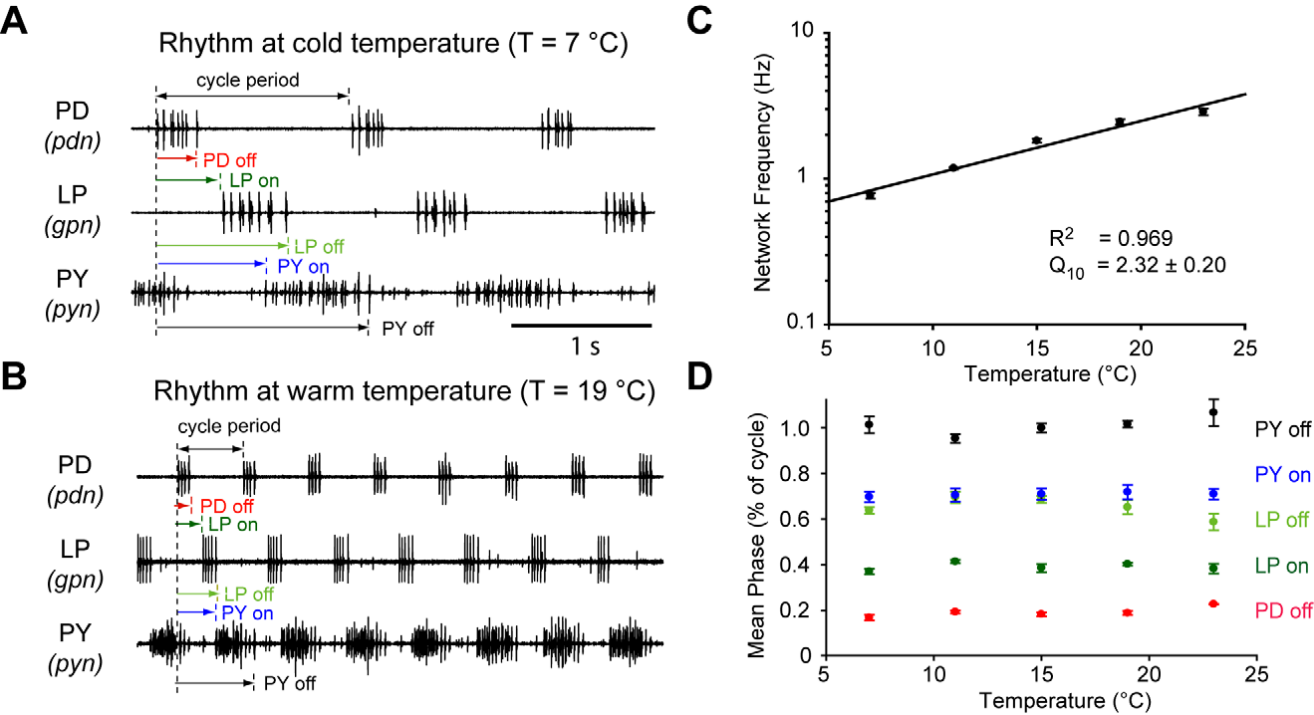
Quantification of pyloric network output at different temperatures. (A) Example extracellular nerve recordings of the pyloric rhythm at cold temperature (T = 7°C). The onset and offset delay of each neuron relative to the onset of PD neuron burst are indicated. Horizontal scale bar, 1 s, for both (A) and (B). (B) Example extracellular nerve recordings from the same preparation as in (A) but at warm temperature (T = 19°C). The same delay measurements are indicated as in (A). (C) The frequency of the pyloric rhythm plotted as a function of temperature from T = 7°C to T = 23°C (*n* = 7). (D) The mean phase (delay divided by cycle period) values of the pyloric rhythm plotted as a function of temperature from T = 7°C to T = 23°C (*n* = 7).

The triphasic pyloric rhythm of the STG is shown in the extracellular recordings from the motor nerves exiting the STG in Figure 1A. The top trace shows a burst of the PD neurons, the second trace shows the activity of the LP neuron, and the bottom trace shows the activity of the PY neurons. By convention, we call the beginning of the PD neuron burst the start of the pyloric rhythm cycle, and the other neurons are referenced to the PD neuron activity. One cycle period is defined as the time between the start of one PD burst and that of the subsequent PD burst. The phases at which each neuron burst starts and ends are defined as the delays to those events divided by the cycle period.

All of the animals used in this study were acclimated to 11°C for at least 3 wk before use. The frequency of the pyloric rhythms from 23 animals at 11°C varied from 1.0 Hz to 1.5 Hz and exhibited a mean frequency of 1.20±0.11 Hz (S.D.). The median pyloric frequency of these acclimated animals was not significantly different from that of a non-acclimated population described previously (*p* = 0.35, Mann-Whitney rank sum test, *n* = 45 for the non-acclimated population) [15]. Interestingly, the variance of pyloric frequency in the acclimated population was lower than that in the non-acclimated population (S.D. = 0.21, *p*<0.05, Levene test).

To quantify the effects of temperature on frequency and phase of the pyloric rhythm, we recorded from the motor nerves in seven preparations that were gradually warmed from 7°C to 23°C (Figure 1). Figure 1A shows the pyloric rhythm at 7°C, and Figure 1B shows recordings from the same preparation at 19°C. Note that the frequency increased substantially, but the relative timing of the triphasic pattern of activity was largely preserved. Figure 1C presents the data pooled from the seven experiments. Over this temperature range the frequency increased about 4-fold from 0.7±0.1 Hz to 2.9±0.4 Hz (S.D.), with a Q_10_ of 2.32±0.2 (Table 1). Temperature had a significant effect on pyloric frequency (*p*<0.01, one-way repeated measures ANOVA, *n* = 7). Remarkably, although the frequency varied considerably as a function of temperature, the phase of firing of the pyloric neurons was virtually unaltered by temperature (Figure 1D, *n* = 7), as indicated by Q_10_'s not significantly different from 1 (Table 1). We did not find evidence for hysteresis as there was no statistically significant difference between increasing temperature from 7 to 23°C versus decreasing temperature from 23 to 7°C (*p* = 0.410, two-way repeated ANOVA, *n* = 4).

**Table 1:** Temperature dependence of pyloric network parameters.

Level	Process	Q_10_	S.E.	m≠0?	*p* Value
*Network*	Cycle Frequency	2.32	0.20	yes	<0.001
*Phase Relationships*	PD off	1.02*	0.07	no	0.083
	LP on	1.00*	0.04	no	0.887
	LP off	0.94*	0.05	no	0.322
	PY on	1.01*	0.01	no	0.090
	PY off	1.04*	0.03	no	0.223
*Synaptic*	IPSPs onto LP neuron	0.92*	0.10	no	0.256
	IPSCs onto LP neuron	2.29	0.25	yes	<0.001
*LP Membrane*	g_A_	1.90	0.44	yes	<0.001
*Currents*	I_A_ activation rate	3.00	0.16	yes	<0.001
	I_A_ inactivation rate	3.78	0.18	yes	<0.001
	g_h_	3.13	0.46	yes	<0.001
	I_h_ activation rate	3.36	0.18	yes	<0.001
*Input Conductance*		1.56	0.17	yes	<0.001

*These Q_10_'s were highlighted for their low temperature sensitivities (i.e., Q_10_∼1). To test whether the slopes (m) of these Q_10_'s were significantly different than zero, *t* tests and their associated *p* values are reported in the right-hand columns.

### The Effects of Temperature on Membrane Potential Trajectories

**Figure 2:**
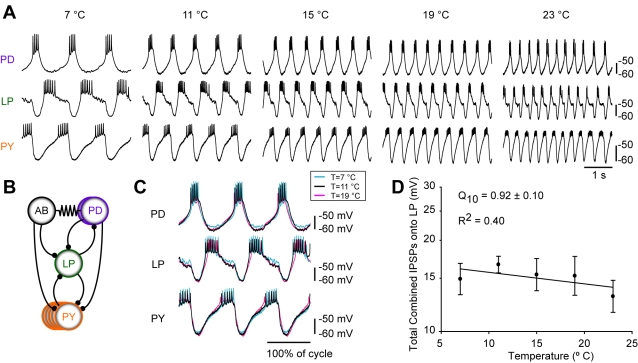
Similarity of membrane potential trajectories and IPSPs of the pyloric neurons at different temperatures. (A) Simultaneous intracellular recordings of PD, LP, and PY neurons of the pyloric rhythm at different temperatures (T = 7, 11, 15, 19, and 23°C, respectively). Vertical scale bar, −60 mV to −50 mV. Horizontal scale bar, 1 s. (B) Simplified diagram of the pyloric circuit. The pacemaker kernel is comprised of the AB neuron and two electrically coupled PD neurons. The follower cells include a single LP neuron and several electrically coupled PY neurons. Filled circles represent inhibitory chemical synapses; resistor symbols represent electrical coupling. (C) Overlays of 3 cycles of PD, LP, and PY neuron activity recorded intracellularly at three different temperature (T = 7°C, T = 11°C, T = 19°C; blue, black, pink, respectively) from the same preparation. These traces were scaled for cycle period and then superimposed upon one another. Vertical scale bar, 10 mV. Horizontal scale bar, 1 duty cycle. (D) Total IPSPs recorded in LP as a function of temperature from T = 7°C to T = 23°C (*n* = 7).

To gain further insight into how phase relationships might remain stable despite the increase in frequency as a function of temperature, we examined the intracellular waveforms of the pyloric neurons as a function of temperature. Figure 2A shows simultaneous intracellular recordings from the PD, LP, and PY neurons in a single preparation at temperatures from 7°C to 23°C (circuit diagram; Figure 2B). Again, while the frequency dramatically increased, the characteristic triphasic motor pattern was maintained, and the intracellular waveforms were similar at all temperatures. This can be seen most effectively by scaling the membrane potential trajectories of the intracellular waveforms to the cycle period (Figure 2C). The membrane potential trajectories of the pyloric neurons are very similar when they are temporally scaled.

Figure 2B shows a simplified connectivity diagram for the pyloric rhythm, and illustrates that the LP neuron is inhibited by the pacemaker neurons and the PY neurons. The phase of LP firing is known to depend both on its synaptic inputs (Figure 2B) and on its intrinsic, voltage-dependent membrane currents [13],[14],[17],[18],[27],[28],[29]. The effects of temperature on the Inhibitory Postsynaptic Potentials (IPSPs) recorded in the LP neuron are plotted in Figure 2D. Note that this is a measure of the combined, total IPSP, consisting of the IPSP evoked first by the PY neurons, followed by the IPSPs evoked by the AB and PD neurons (Figure 2C). We measured the total IPSP as the amplitude of the membrane potential excursion from the end of the LP burst to its most hyperpolarized value (the first component comes from the PY neurons, and the second sharp hyperpolarization from PD/AB neuron activity). The total IPSPs recorded in LP were not statistically different over the temperature range shown (Q_10_ = 0.92±0.1, *p* = 0.256, one-way repeated measures ANOVA; Table 1).

### Effects of Temperature on Synaptic Currents

**Figure 3:**
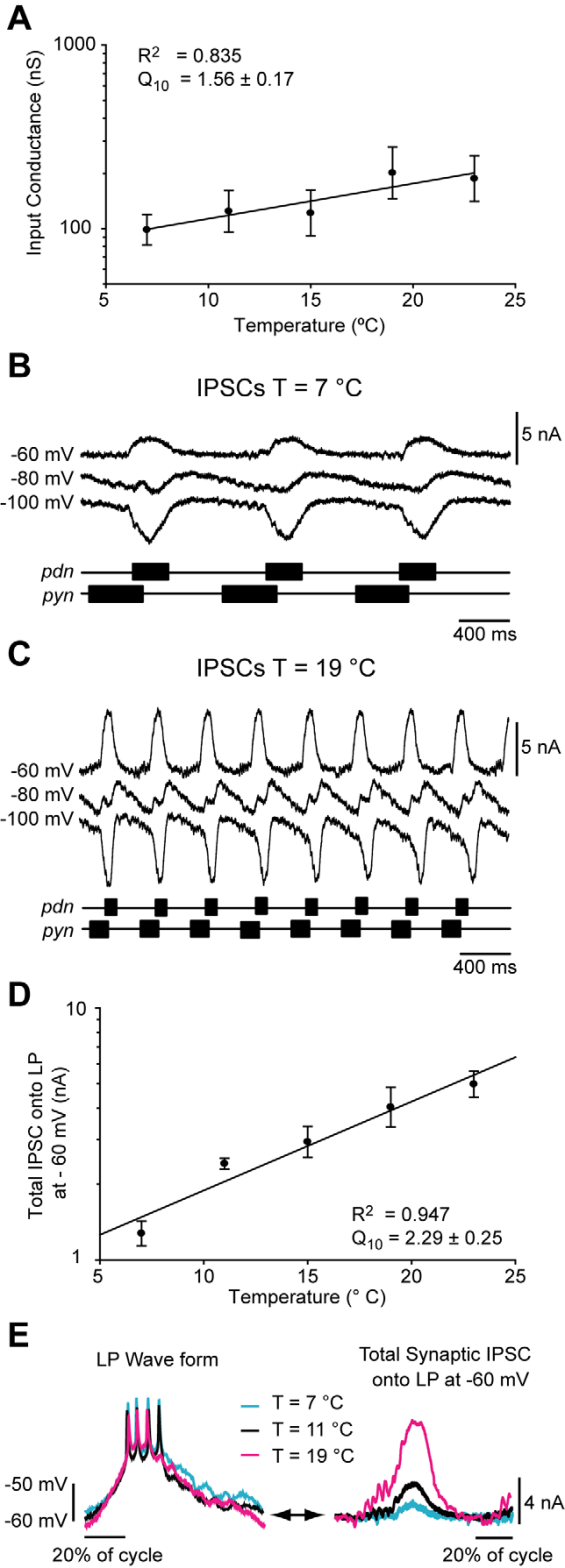
Input conductance and IPSCs as a function of temperature. (A) Input conductance of the LP neuron as a function of temperature was measured in the presence of 10^−7^ M TTX and 10^−5^ M PTX in the passive range (−60 to −80 mV). (B) Example IPSCs of the LP neuron recorded at cold temperature (T = 7°C) at holding potentials of −60, −80, and −100 mV. The corresponding extracellular recordings of the pyloric dilator nerve (*pdn*) and pyloric nerve (*pyn*) showing the corresponding PD and PY bursts are shown schematically. Vertical scale bar, 5 nA. Horizontal scale bar, 400 ms. (C) Example IPSCs from the same LP neuron as in (A) but at warmer temperature (T = 19°C). (D) Amplitude of the total IPSC at −60 mV as a function of temperature from T = 7°C to T = 23°C (*n* = 7). (E) *Left:* Overlay of LP waveform at temperatures of 7, 19, and 23°C from the same preparation. Horizontal scale bar, 20% duty cycle. Vertical scale bar −60 to −50 mV. Right, overlay of the corresponding total IPSC of the LP neuron at −60 mV at temperatures of 7, 19, and 23°C. Horizontal scale bar, 20% duty cycle. Vertical scale bar, 4 nA.

Although the total IPSPs in LP are temperature compensated, this does not necessarily mean that the synaptic currents are also constant, as the IPSP waveform and amplitude depend on the other membrane conductances and time constants as well as the synaptic current. We measured the LP neuron input conductance in voltage clamp as a function of temperature by applying tetrodotoxin (TTX) and picrotoxin (PTX) to remove action potentials and block most of the synaptic inputs to the LP neuron. We then stepped the voltage from −60 mV to −80 mV in 5 mV steps, measured the resulting currents, and calculated the input conductance. Figure 3A shows that the input conductance increased with temperature (*p*<0.002, one-way repeated measures ANOVA), with a Q_10_ of 1.56±0.17.

The apparent lack of change in IPSP amplitude from 7°C to 23°C despite a nearly 2-fold increase in input conductance over that range was intriguing. Therefore, we voltage-clamped the LP neuron during the ongoing pyloric rhythm and directly measured the total Inhibitory Postsynaptic Currents (IPSCs) over the same range of temperatures. Figures 3B and 3C show the IPSCs measured at 7°C and 19°C at three different voltages, −60 mV, −80 mV, and −100 mV. The timing of PY and PD neuron activity is shown below the IPSC recordings so that the PY and AB/PD components of the IPSCs can be seen. At −60 mV at 7°C there was little or no net current seen during PY time, but the current was clearly outward during PD time. At 19°C a small outward current is observed during PY time and a much larger outward current during PD time. The waveforms seen at −80 mV are complex because the PY and AB glutamatergic IPSCs reverse at a more depolarized potential than does the cholinergic PD IPSC [15],[30]. At −100 mV, all components were inward.

The synaptic currents recorded at −60 mV are most relevant to understanding the membrane potential trajectories seen during the ongoing rhythm (Figure 2A,C). Accordingly, the total IPSCs measured at −60 mV as a function of temperature are plotted in Figure 3D. These exhibit a Q_10_ of 2.29±0.25 (*n* = 7). Figure 3E shows superimposed membrane potential trajectories at three temperatures, and their corresponding IPSCs recorded from the same preparation (at −60 mV). Together, these data illustrate that despite the virtually superimposable voltage trajectories, the underlying IPSCs are almost 5-fold larger at the higher temperatures. Moreover, it appears that this increase would more than compensate for the modest increase in input conductance over the same range (Q_10_ = 1.56±0.17; Figure 3A). This suggests that the constant waveform can only partially be accounted for by the changes in input conductance and IPSC amplitude.

### Effects of Temperature on I_A_ and I_h_

Previous work demonstrated the importance of both the transient outward current, I_A_, and the hyperpolarization-activated inward current, I_h_, on the phase at which the LP and PY neurons recover from inhibition [15],[16],[17],[27]. Consequently, we measured the effects of temperature on these currents in voltage clamp, as we hypothesized that changes in these currents could contribute to the temperature compensation of phase (Figure 1D).

**Figure 4:**
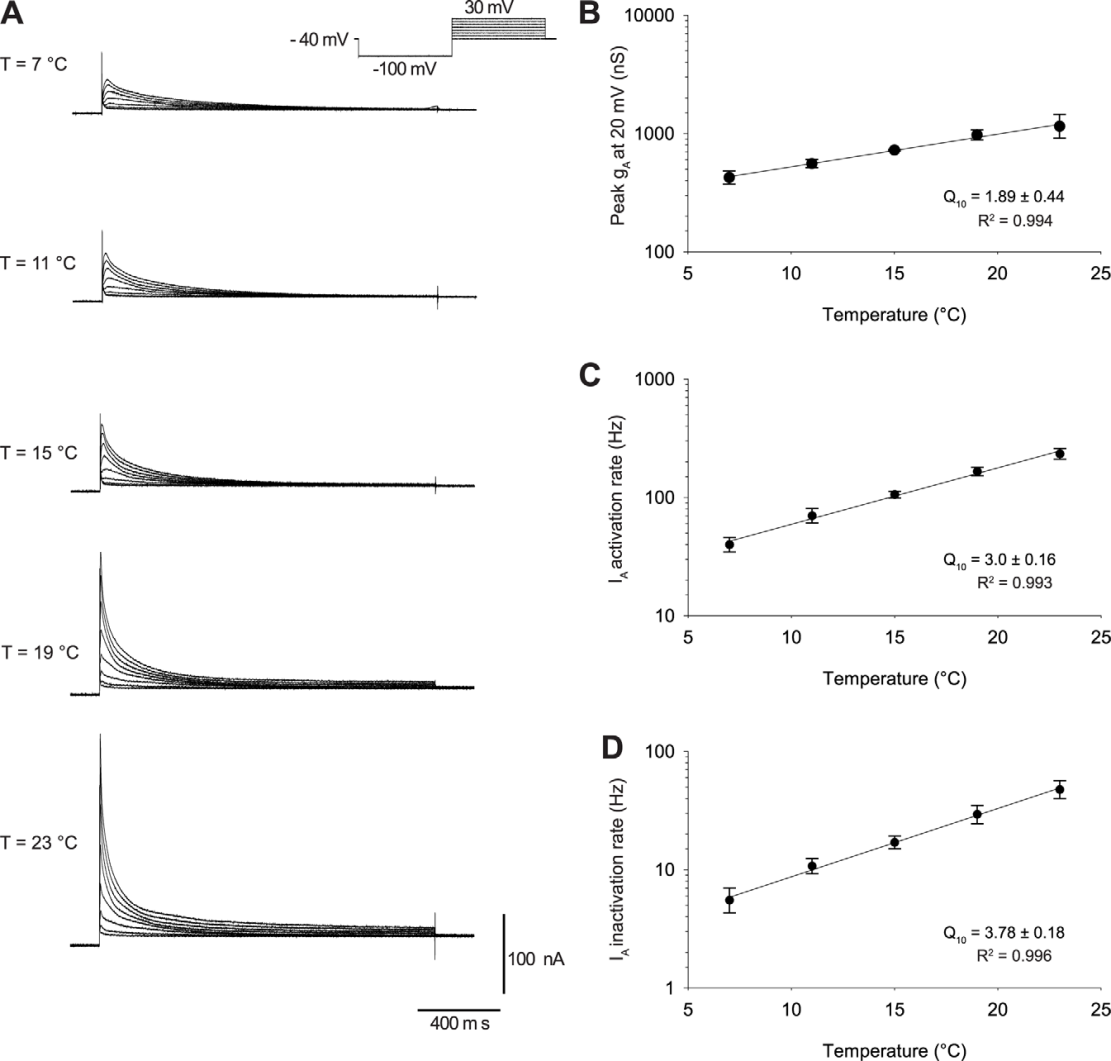
Temperature dependence of I_A_ conductance, activation rate, and inactivation rate. (A) Family of I_A_ currents at 7, 11, 15, 19, and 23°C elicited in response to depolarizing steps from −40 mV to +30 mV in 10 mV steps. (B) Pooled data for the temperature dependence of I_A_ peak conductance measured at +20 mV (*n* = 6). (C) Temperature dependence of I_A_ activation rates was measured as the reciprocal of the time to maximal current elicited at +20 mV (from the time of the depolarizing step). (D) Temperature dependence of I_A_ inactivation rates was measured as the reciprocal of the time to decay to half of the maximal current from the time of maximal current elicited at +20 mV.

Figure 4A illustrates the effects of temperature on I_A_. I_A_ was measured as the difference current between the outward currents evoked from a holding potential of −100 mV and those evoked from −40 mV. The currents measured at voltages from −40 mV to +30 mV in 10 mV steps are shown at five temperatures from 7°C to 23°C (Figure 4A). As the temperature was increased, the amplitude of the peak outward current increased (Figure 4B). The activation rate as well as the inactivation rate of I_A_ also increased as a function of temperature (Figure 4C and 4D, respectively). The Q_10_ of the I_A_ inactivation rate was 3.78, the steepest temperature dependence of the parameters we measured (Table 1).

**Figure 5:**
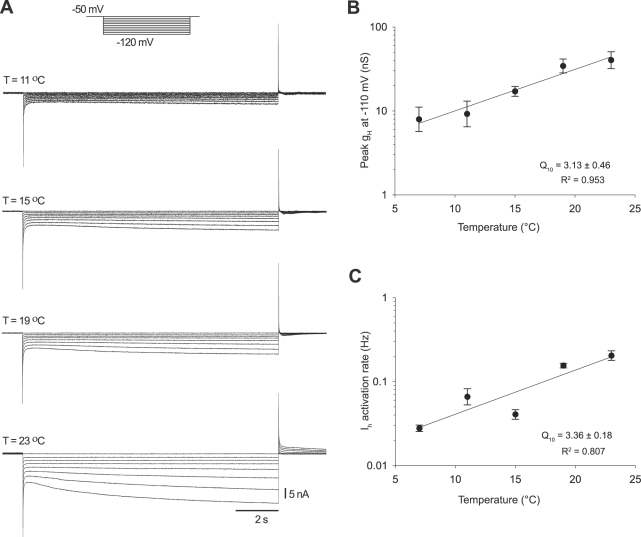
Temperature dependence of I_h_ conductance and activation rate. (A) Family of I_h_ currents at 11, 15, 19, and 23°C elicited in response to hyperpolarizing steps from −50 mV to −120 mV in 5 mV steps from a holding potential of −50 mV (10 mV steps shown here). (B) Pooled data for the temperature dependence of I_h_ peak conductance measured at −110 mV (*n* = 7). (C) Temperature dependence of I_h_ activation rates were measured as the reciprocal of the activation time constants obtained from single exponential fits of the current at −110 mV (*n* = 7).

Lastly, we measured the temperature dependence of I_h_. Because I_h_ is a depolarizing current that is activated only at hyperpolarized potentials, its properties allow I_h_ both to respond to inhibitory synaptic currents and to play an opposing role to I_A_ in determining the latency to firing after synaptic inhibition. We measured I_h_ using 12 second hyperpolarizing voltage steps from −50 mV to −120 mV in 5 mV steps (Figure 5A) at different temperatures. The amplitude of the current (Figure 5B) as well as its activation rate increased at higher temperatures (Table 1).

### The Effects of Temperature on Models of a Bursting Pacemaker Neuron

**Figure 6:**
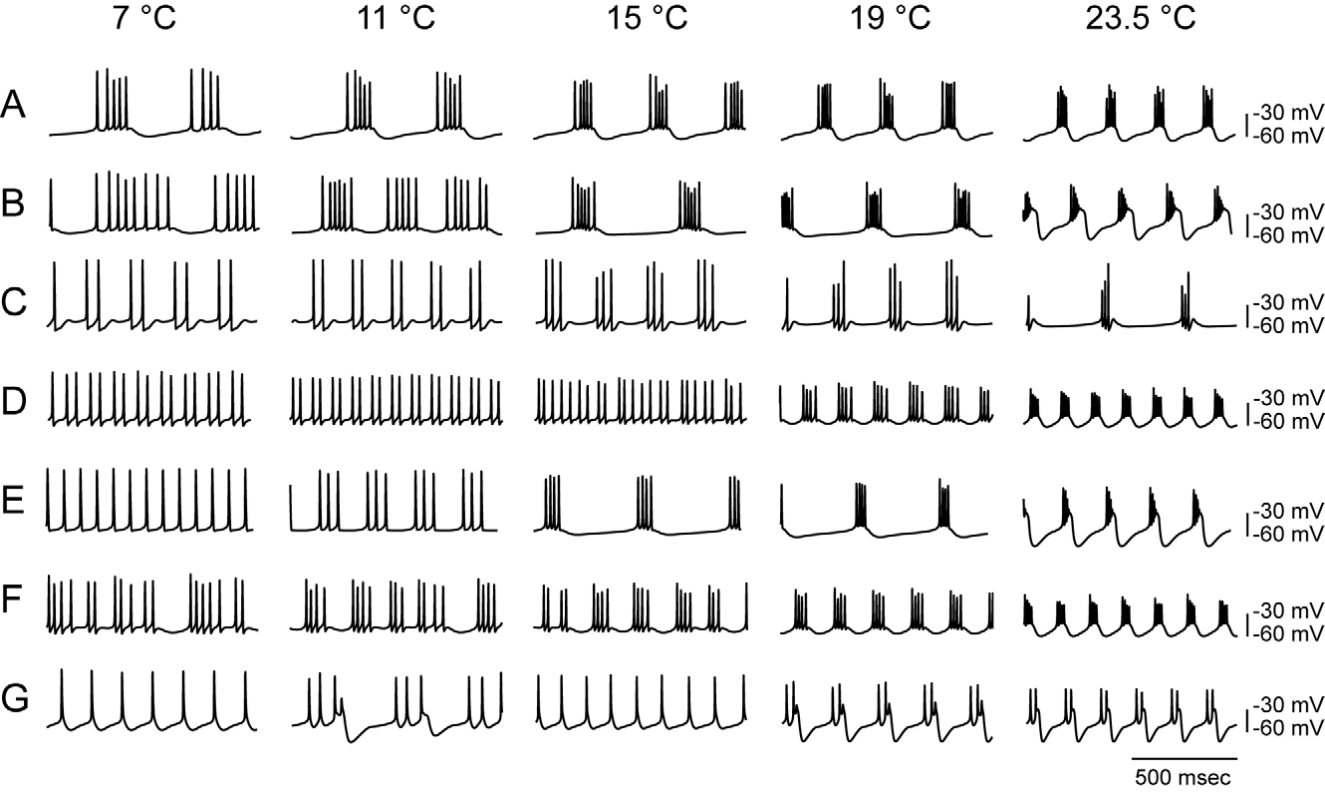
Diverse electrical behavior of model bursting neurons in response to changing temperature. Examples that illustrate the diversity of firing patterns of simulated model neurons in response to changing temperatures from 23.5°C to 7°C. (A) Neuron that increases burst frequency as a function of temperature. (B) Neuron that decreases burst frequency sharply from 11 to 15°C, then subsequently increases with temperature. (C) Neuron that decreases burst frequency from 11 to 23.5°C. (D) Neuron that transitions from tonic spiking to bursting behavior at 19°C. (E) Neuron that transitions from tonic spiking at 11°C, then increases burst frequency with increase in temperature. (F) Neuron that transitions from irregular spiking to bursting behavior. (G) Neuron that switches its firing pattern twice: the neuron tonically spikes at 7°C, bursts at 11°C, reverts to spiking at 15°C, and then switches to bursting at 19 and 23.5°C.

Temperature increased the frequency of the pyloric rhythm (Figure 1). The membrane properties of the PD/AB pacemaker group are the most important determinant of pyloric frequency [31],[32],[33]. Consequently, we were interested to determine whether increases in frequency with increasing temperature in bursting neurons are to be generally expected. Therefore, we studied the effects of temperature on model bursting neurons [25], whose currents were initially based on those recorded from lobster STG neurons [34]. We first generated 1270 models, all of which were bursting at 23.5°C, but which differed in the maximal conductances of their 7 membrane currents. We then changed the temperature of all of the models, using a Q_10_ of 2 for all of the activation and inactivation rates of the currents. Figure 6 shows the effects of temperature on 7 of the 1,270 models studied and illustrates that models with differing sets of maximal conductances varied dramatically in their response to temperature.

Only 60.5% of the models showed bursting over the entire range of temperatures and increased monotonically in frequency over the full temperature range. Of this 60.5%, 68.5% maintained approximate constant duty cycle over the entire temperature range, a feature that would promote phase compensation in the network.

The remaining 39.5% of the models either did not burst over the entire range or displayed decreases in frequency over at least some of the temperature range (Figure 6B–G). Together these data indicate that the maximal conductances of the currents are important in determining how that particular neuron will behave in response to a temperature change.

### The Effects of Temperature on Phase Compensation in Model LP Neurons

**Figure 7:**
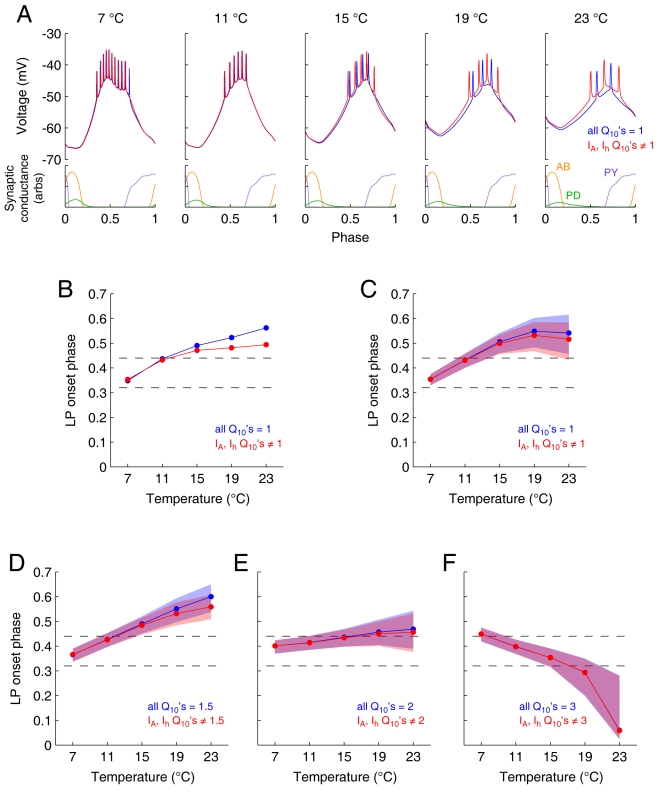
Effects of temperature on a population of 1304 LP models. (A) Voltage traces of an example LP model at different temperatures, for a single cycle of simulated pyloric synaptic input. Blue traces are for a model with all Q_10_'s set to one. Red traces are the same model, but with the Q_10_'s for I_A_ and I_h_ set to their measured values. Lower panels show the synaptic conductances injected into the model. At each temperature, the synaptic input had a frequency as given by the linear fit in Figure 1C. The *x*-axis in each panel has been scaled to show the voltage versus phase. (B) LP onset phase versus temperature for the same model as shown in (A). Dashed lines here and in other panels show the bounds of the central ∼85% of the distribution of LP phase onsets observed experimentally [15]. (C) LP onset phase versus temperature for all 1,304 models. Line shows the median LP onset phase for all models that fired at that temperature. Shaded region shows the range of the 25th to 75th percentile. (D) Blue simulations had a Q_10_ of 1.5 for all intrinsic conductances, and the red simulations had the Q_10_'s for I_A_ and I_h_ set to their measured values. (E) A “default” Q_10_ of 2.0 for all intrinsic conductances. (F) Default Q_10_ of 3.0. Note that (A), (B), and (C) are not directly comparable with (D), (E), and (F), because (A), (B), and (C) had a Q_10_ of 1.0 for the synaptic conductances, whereas (D), (E), and (F) used the experimentally measured Q_10_ of 2.3 for the synaptic conductances.

Taylor et al. [26] recently developed a family of 1304 LP neuron models, each of which has different values of its maximal conductances and all of which fit a set of criteria matching the biological LP neuron. We used this set of LP neuron models to ask whether the temperature dependence of I_A_ and I_h_ contribute to temperature compensation of the LP neuron's phase. Figure 7A shows the results of a simulation in which we varied the temperature of a model LP neuron. In this set of simulations, we compared two conditions: (a) all model Q_10_'s were set to 1, and (b) the Q_10_'s of I_A_ and I_h_ were replaced by the experimentally measured values (Figures 4 and 5). The LP onset phase typically became more delayed as the frequency was increased (Figure 7A,B,C; *p*<0.0001, Kruskal-Wallis test, *n* = 1,063), but the biologically measured Q_10_'s decreased this effect (*p*<0.0001, sign test on 23°C data, *n* = 1,063) and increased the extent to which the LP maintained constant phase as a function of temperature.

We then examined the effects of changing all of the model Q_10_'s to 1.5, 2.0, and 3.0 (Figure 7D,E,F, respectively) and again compared the effects of using these default Q_10_'s for I_A_ and I_h_ (blue) to the condition in which they had the experimentally determined values (red). These plots show that in the “default” (blue) condition, temperature affected the LP onset phase for all Q_10_'s (*p*<0.0001 for all three Q_10_'s, Kruskal-Wallis test, *n* = 1,148, 1,255, and 1,109, respectively). For the blue condition, LP onset phase came later at high temperatures for Q_10_ = 1.5, became approximately temperature compensated for Q_10_ = 2.0, and became considerably “overcompensated” for Q_10_ = 3.0 (slopes were significantly different, *p*<0.0001, Kruskal-Wallis test, *n* = 1,012). Note that the differences between the red and blue conditions were smaller for larger Q_10_ values (at 23°C, there was a significant effect of Q_10_ on the difference between red and blue conditions, *p*<0.0001, Kruskal-Wallis test, *n* = 1,138; all pairwise differences between Q_10_'s were significant at *p*<0.0001, rank sum test with Bonferroni correction).

## Discussion

Poikilotherms often face considerable temperature fluctuations during a given day or over extended periods of time. This poses a series of interesting questions for the nervous system, as it needs to maintain its important functions despite these environmental perturbations. Many previous studies have documented the effects of temperature on a variety of behaviors and physiological processes [3],[35],[36],[37],[38],[39]. Other studies have looked at the effects of temperature on individual biochemical reactions, ion channels, synapses, or other cellular mechanisms [6],[40],[41],[42],[43],[44],[45]. Nonetheless, much less work has been done directly on the effects of temperature on behaviorally relevant neuronal circuits. This study is one of relatively few to attempt to account for the effects of temperature on a neuronal circuit in terms of the cellular processes that contribute to those circuit behaviors.

### The General Problem of Temperature Compensation

By convention, processes with Q_10_'s less than 2 are considered to be relatively temperature insensitive, and behaviors with emergent system Q_10_'s less than 2 are conventionally termed “temperature compensated.” The best known example of temperature compensation is that shown by the circadian rhythm, and its temperature compensation is considered one of its salient features. Although the molecular basis underlying circadian rhythms have been well-elucidated [1], the mechanisms that underlie their temperature compensation remain unresolved. However, a recent study on temperature compensation in *Neurospora* [2] argues that temperature compensation arises from compensatory changes in the rates of phosphorylation and degradation of proteins that are part of the molecular clock.

Zhurov and Brezina [46] have provided an elegant study of a different form of temperature compensation in the *Aplysia* neuromuscular system. In this preparation, the strength of neuromodulatory effects on muscle contraction was maintained over a temperature range from 15°C to 25°C. Paradoxically, the release of neuromodulators that modulate neuromuscular contraction decreased 20-fold when temperature was increased. However, the decrease in neuromodulator release was partially compensated by increased neuromodulator efficacy. Thus in this particular example, physiological temperature compensation is achieved by opposing temperature dependencies of related functions.

In this study, we describe a form of temperature compensation that occurs in a rhythmically active neural circuit. When we rapidly varied the temperature of the in vitro stomatogastric nervous system, the pyloric network frequency varied about 4-fold, from ∼0.7 Hz at low temperatures to ∼2.9 Hz at 23°C (Figure 1C). At the same time, the phase relationships of the pyloric rhythm were tightly maintained over this large frequency range (Figure 1). The almost perfect temperature compensation of pyloric rhythm phase (Table 1) described here surpasses that described in many systems, including some circadian rhythms [2],[46].

**Figure 8:**
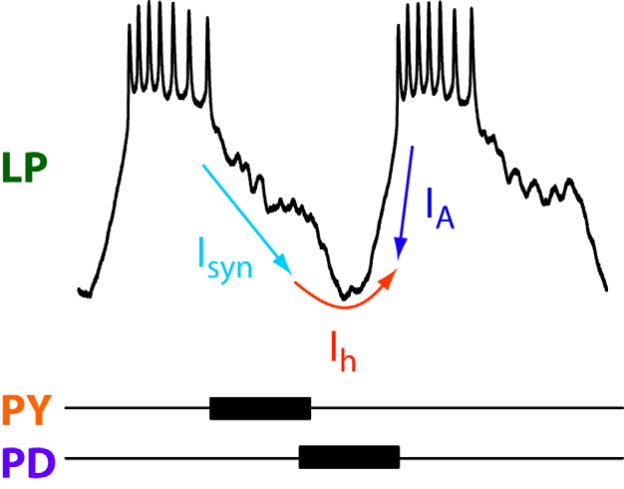
Schematic of the processes that contribute to the control of phase in the LP neuron. LP neuron activity is terminated by inhibitory synaptic input (light blue arrow) and the onset of this synaptic input is initiated by the PY neurons (orange). PY neuron activity continues until the PD/AB neurons start to fire (purple), at which time the PD/AB neurons strongly inhibit the LP neuron. After the end of the PD/AB burst, the LP starts firing with a delay that is decreased by activation of I_h_ (red arrow) and increased by the activation of I_A_ (dark blue arrow).

All of the component processes we measured increased in amplitude and/or rate with temperature. However, given that some of these component processes—e.g., I_A_ and I_h_—have opposing functional roles, temperature compensation of the phase relationships of the pyloric neurons appears to emerge as a consequence of similar temperature dependencies of opposing cellular mechanisms. Figure 8 shows a cartoon of the main processes that are likely to control the phase of the LP neuron's activity. LP's activity is terminated by PY neuron activity, and then additionally inhibited by the AB/PD neurons during the pacemaker burst. Rebound firing after the AB/PD burst is enhanced by I_h_ activation and delayed by I_A_ activation [17],[27]. An increase in the IPSC amplitude will tend to increase the activation of I_h_ and also favor the voltage-dependent deinactivation of I_A_. Consequently, as temperature increases, these opposing time and voltage-dependent processes, as a first approximation, may remain temporally scaled with each other. The LP off-phase is determined by the phase of PY onset, which is itself maintained constant, presumably by similar mechanisms.

### Phase and Frequency in Motor Systems

At constant temperature, the pyloric network phase relationships are also maintained despite changes in frequency [13],. This has been extensively studied because it is not obvious how to turn the constant delays associated with the fixed dynamics of synaptic currents or other membrane currents into a mechanism that confers phase constancy. Again, as illustrated in Figure 8, it has been suggested that the latency to firing of a follower neuron is controlled by multiple processes, including synaptic depression, I_A_, and I_h_ [20],[48]. At constant temperature, the frequency dependence of synaptic depression of the AB/PD IPSP is balanced by the time-dependence of activation of I_h_ and deinactivation of I_A_ [24].

Thus, the mechanisms that allow for phase maintenance at constant temperature seem to employ some of the same cellular mechanisms that promote temperature compensation. This is not necessarily surprising, because the strong evolutionary pressure that gave rise to robust phase maintenance may have also forced a set of solutions that would be robust enough to compensate for the frequency changes that arise from changes in temperature.

The ability to maintain phase independent of frequency has been well-described in the neuronal control of swimming in lamprey, leech, and crayfish [49],[50],[51]. In these systems, phase is constant over a wide range of frequencies [49],[50],[51], and it would be interesting to see how well temperature compensated they are. It is surprising that in the zebra finch, a homeotherm, in which singing behavior is highly stereotyped, cooling specific brain areas in the motor pathway leads to uniform elongation of the song [52]. We speculate that the strong temperature dependence of frequency and temperature-independence of phase may not be unique to the pyloric circuit of the stomatogastric nervous system and may be a useful property to other animals, allowing them to cope with environmental challenges in their natural setting.

### The Effects of Temperature on Frequency and Phase in Computational Models

Our commonsense knowledge of the world may lead us to believe that achieving robust behavior in cold-blooded animals over a range of temperature is simpler than it is in fact. The non-trivial nature of this problem is revealed in the computational studies reported here. In both cases, we employed families of more than 1,000 model neurons that varied in terms of the maximal conductances of their underlying currents but were otherwise identical. These modeling studies illustrate the extent to which robust physiological behavior requires that the temperature dependence of the biological processes must be closely regulated.

Many of the 1,270 pacemaker models (Figure 6) failed to maintain a bursting phenotype over the entire temperature range or else showed interesting non-monotonic changes in frequency. In some cases bursting frequency decreased as temperature increased between certain temperature points (Figure 6B and E) or along the entire temperature range (Figure 6C). One implication of this model is that not all combinations of currents that give rise to bursting at one temperature will necessarily provide robust behavior at other temperatures. Furthermore, the requirement that a neuron or group of neurons show increased burst frequency in a clean monotonic fashion as temperature is increased may considerably restrict the parameter space in which “good enough” solutions may reside.

Although there has been a good deal of both experimental and theoretical work on the importance of both I_A_ and I_h_ in determining phase in the pyloric network, without the computational studies shown in Figure 7, it was difficult to know whether the specific Q_10_'s of I_A_ and I_h_ would favor temperature compensation of phase. The first result from these studies (Figure 7C) was that the measured Q_10_'s of I_A_ and I_h_ promote temperature compensation of phase. This effect was most pronounced when it was examined with a background of low Q_10_'s of the other currents in the models (Figure 7C,D). When the background Q_10_'s were higher (Figure 7E,F), although I_A_ and I_h_ still statistically altered the phase onset of the LP neuron, the extent of their influence decreased.

Taken at face value, the data we obtained with the 1,304 LP neuron models (Figure 7) suggest that temperature compensation of phase will be optimized when the Q_10_'s of all of the currents that contribute to phase are close to the Q_10_ of the pyloric rhythm frequency, which was ∼2.3 (Figure 1).

### General Implications

Although it is possible for the same neuron in different animals to have widely disparate sets of current densities for ion channels [15],[53],[54], the Q_10_'s of these currents and other biological processes must be presumably regulated within a certain range. The actual number of enzyme molecules or ion channels in a given neuron in an individual animal may vary widely according to the life history of the animal. On the other hand, the temperature-dependence of biological enzymes and ion channels has arisen as a result of evolution working on the structure of each protein molecule, so one would naively predict that the Q_10_'s of a given process measured across animals of the same species might vary less than the number of ion channels or enzyme molecules. Of course, post-translational and other history-dependent processes could also influence the Q_10_'s of many biological processes, so the process of producing both robust behavior and temperature compensation over a large temperature range requires the coordinated regulation of myriad biological processes.

## Methods

### Animals

*Cancer borealis* were purchased from Commercial Lobster (Boston, MA) and maintained in tanks containing artificial seawater at 11°C for 3 wk before use.

### Solutions

*C. borealis* physiological saline was composed of 440 mM NaCl, 11 mM KCl, 13 mM CaCl_2_, 26 mM MgCl_2_, 11 mM Trizma base, and 5 mM Maleic acid, pH 7.4–7.5. All reagents were purchased from Sigma Aldrich.

### Electrophysiology

The stomatogastric nervous system was dissected out of the animals and pinned out in a Sylgard (Dow Corning) coated plastic Petri dish containing chilled saline (11°C). In all cases, we worked only with fully intact stomatogastric nervous system preparations that included the commissural and esophageal ganglia with two intact superior esophageal nerves and two intact inferior esophageal nerves.

The preparations were continuously superfused with physiological saline. The temperature of the superfusing saline was controlled using a Peltier device purchased from Warner Instruments. The temperature was changed at about 1 degree/min when only extracellular recordings were made and about 1 degree/2–3 min when intracellular recordings were also made. As the temperature was increased we noticed that the ganglia swelled and/or moved. This often necessitated small movements of the intracellular electrodes (up or down, respectively, as the temperature was increased or decreased) to maintain the intracellular recordings. When the electrodes were repositioned carefully, return to 11°C showed no changes in resting potential, firing properties, or input resistance. If the electrodes were not repositioned slightly, penetrations were usually lost with large temperature changes. The preparations were maintained at their target temperature for 10 min before data were taken.

Vaseline wells were placed around motor nerves and extracellular recordings were obtained using stainless steel pin electrodes placed in the wells and amplified using an A-M Systems differential amplifier. Intracellular recordings were obtained from cell bodies in the STG using 10–30 MΩ glass microelectrodes pulled with a Flaming/Brown micropipette puller (Sutter Instrument, Co.) The microelectrode solution contained 0.6 M K_2_SO_4_ and 20 mM KCl. Data were acquired using a Digidata 1200 data acquisition board (Axon Instruments) and analyzed using Clampfit 9.0 (Axon Instruments) Spike2 v 6.04 (Cambridge Electronic Design) and/or MATLAB 7.1 (Mathworks). Statistical analyses were performed using the SigmaPlot 10 and SigmaStat software packages (Jandel Scientific).

Input conductance was measured in two electrode voltage clamp by stepping the membrane from −60 mV to −80 mV in 5 mV steps, measuring the resulting currents, and then calculating the conductance.

IPSCs were measured using two electrode voltage clamp during the ongoing rhythm. IPSCs were measured in the LP neuron at membrane potentials from −60 mV to −120 mV in 5 mV steps. Each holding potential was maintained for 8–12 s, and the membrane potential was returned to −60 mV in between each voltage step for at least 8–12 s.

I_h_ and I_A_ were both measured in the presence of 10^−7^ M TTX, 10^−5^ M PTX, and 10^−2^ M TEA. I_h_ was measured at membrane potentials from −50 mV to −120 mV in 5 mV steps using 12 s pulses with 12 s at −50 mV between each pulse. I_A_ was measured as the difference current between steps taken from a holding potential of −100 mV and those taken from a holding potential of −40 mV. Currents were recorded from −40 mV to +30 mV at 10 mV intervals using pulses of 1.5 s following a 1.5 s prepulse.

### Q_10_ Calculations

To characterize and compare the temperature dependencies of various parameters of the pyloric network, we used a modified Arrhenius equation to determine the temperature coefficient, Q_10_. The Q_10_ is a conventional measure used to describe how much a given rate process changes over a 10°C temperature change and defined as [rate(T+10°)/rate(T)] [55]. A given parameter *p* was plotted against temperature T in a semilog plot and the Q_10_ value was then extracted from the slope of the linear regression (*m*) [3].
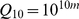
(1)

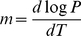
(2)The standard error of the Q_10_ (*se*_Q10_) was calculated by propagation error from the slope and the standard error of the slope (*se*_m_) of the linear regression [56]:
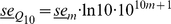
(3)Although the alternative Arrhenius activation energy values are implicit in our analyzed data, we did not express this more physical description due to the non-unitary nature of our parameters (i.e., they are not simple processes with a single rate-limiting barrier).

### The Bursting Pacemaker Models

The model used to simulate bursting neurons has been previously described [25]. Briefly, this contains seven active voltage-dependent conductances, a leak conductance, and an algorithm for activity-dependent homeostatic regulation of maximal channel conductances. We added to this model the ability to control the temperature sensitivity (Q_10_) of all channel activation and inactivation dynamics. For simplicity we set the Q_10_ of all the activation and inactivation rates equal to 2. All values for *m_∞_*, *h_∞_*, *τ_m_*, *τ_h_*, and *p* are as found in Liu et al. [25] but modified to incorporate the Q_10_.

To create a population of model bursting neurons, we initiated 1,500 simulations with random initial values for all seven active conductances. Each model was subject to 1 h of simulated activity-dependent homeostatic regulation at the model's default temperature of 23.5°C. Models that showed poor bursting behavior after this period were excluded, resulting in a population of 1,270 model neurons that were bursting at 23.5°C and which had a variety of different values of the maximal conductances for the voltage-dependent currents. We then turned off the self-tuning function in these neurons and used them as a set of models with different underlying conductances to probe the extent to which they maintained functional bursting activity over a wide range of temperatures.

Each model was run at 7, 11, 15, and 19°C—the same temperatures used in the biological studies. Voltage traces from each of these simulations were analyzed to determine how the bursting behavior varied with temperature. Bursting behavior—as measured by slow wave frequency—was extracted from each simulation using auto-correlation of a 10 s sample of the voltage trace. Here we define slow wave frequency as low-frequency membrane oscillations. We identified the frequency of these oscillations as the lowest frequency peak in the autocorrelogram of the voltage waveform. If this peak had an autocorrelation less than 0.05, we instead used the frequency corresponding to the greatest autocorrelation (this is uncommon and corresponds to very irregular activity).

To determine whether the bursting neurons maintained duty cycle as a function of temperature, we calculated the coefficient of variation (CV) of each model's duty cycle. The bounds used to define the population of model bursting neurons that exhibited “constant duty cycle” were chosen to contain the central ∼85% of the experimental data (PD off phase). Models exhibiting CVs of duty cycle across temperature within this bound were classified as having constant duty cycle, while those with CVs above this value were classified as having a “non-constant duty cycle.”

### The LP Neuron Models

Simulations to determine how the Q_10_'s of I_A_ and I_h_ affect LP phase onset were performed using the LP model described in Taylor et al. [26]. This model was modified so that Q_10_'s could be associated with all voltage-gated and synaptic conductances. For each voltage-gated conductance, there was a Q_10_ for the maximal conductance, another for the activation rate, and another for the inactivation rate. The reference temperature was 10°C, as the model was originally designed to mimic LP's behavior at this temperature. Reversal potentials (and the GHK relation for the calcium current) were not changed with temperature, as these effects are small over the temperature range used.

## Abbreviations

AB: Anterior Burster
IPSCs: Inhibitory Postsynaptic Currents
IPSPs: Inhibitory Postsynaptic Potentials
PD: Pyloric Dilator
PTX: picrotoxin
PY: Pyloric
STG: stomatogastric ganglion
TTX: tetrodotoxin
